# Automated detection of steps in videos of strabismus surgery using deep learning

**DOI:** 10.1186/s12886-024-03504-8

**Published:** 2024-06-10

**Authors:** Ce Zheng, Wen Li, Siying Wang, Haiyun Ye, Kai Xu, Wangyi Fang, Yanli Dong, Zilei Wang, Tong Qiao

**Affiliations:** 1 Department of Ophthalmology, Xinhua Hospital Affiliated to Shanghai Jiaotong University School of Medicine, Shanghai, China; 2 Department of Ophthalmology, Shanghai Children’s Hospital, School of Medicine, Shanghai Jiao Tong University, Lu Ding Road # 355, Shanghai, 200000 China

**Keywords:** Deep learning, Strabismus surgery, Automated detection, Surgical videos

## Abstract

**Background:**

Learning to perform strabismus surgery is an essential aspect of ophthalmologists’ surgical training. Automated classification strategy for surgical steps can improve the effectiveness of training curricula and the efficient evaluation of residents’ performance. To this end, we aimed to develop and validate a deep learning (DL) model for automated detecting strabismus surgery steps in the videos.

**Methods:**

In this study, we gathered 479 strabismus surgery videos from Shanghai Children’s Hospital, affiliated to Shanghai Jiao Tong University School of Medicine, spanning July 2017 to October 2021. The videos were manually cut into 3345 clips of the eight strabismus surgical steps based on the International Council of Ophthalmology’s Ophthalmology Surgical Competency Assessment Rubrics (ICO-OSCAR: strabismus). The videos dataset was randomly split by eye-level into a training (60%), validation (20%) and testing dataset (20%). We evaluated two hybrid DL algorithms: a Recurrent Neural Network (RNN) based and a Transformer-based model. The evaluation metrics included: accuracy, area under the receiver operating characteristic curve, precision, recall and F1-score.

**Results:**

DL models identified the steps in video clips of strabismus surgery achieved macro-average AUC of 1.00 (95% CI 1.00–1.00) with Transformer-based model and 0.98 (95% CI 0.97-1.00) with RNN-based model, respectively. The Transformer-based model yielded a higher accuracy compared with RNN-based models (0.96 vs. 0.83, *p* < 0.001). In detecting different steps of strabismus surgery, the predictive ability of the Transformer-based model was better than that of the RNN. Precision ranged between 0.90 and 1 for the Transformer-based model and 0.75 to 0.94 for the RNN-based model. The f1-score ranged between 0.93 and 1 for the Transformer-based model and 0.78 to 0.92 for the RNN-based model.

**Conclusion:**

The DL models can automate identify video steps of strabismus surgery with high accuracy and Transformer-based algorithms show excellent performance when modeling spatiotemporal features of video frames.

**Supplementary information:**

The online version contains supplementary material available at 10.1186/s12886-024-03504-8.

## Background

Strabismus, defined as any binocular misalignment, affects 0.8–6.0% of children and can lead to amblyopia, visual impairment, or even hampered visual system’s development if left untreated [1–4]. Strabismus surgery, successfully realigns the eyes by adjusting eye muscle tension or position [5, 6], thereby alleviating double vision and improving quality of life. Competence in strabismus surgery is crucial for ophthalmology residents worldwide, as recommended from the American Board of Ophthalmology (ABO) in the United States [7, 8]. In the United Kingdom [9], the ophthalmic specialty training curriculum requires trainees to have completed 20 surgical strabismus procedures by completion of training. In China, however, ophthalmology residents reportedly perform less surgery during their training [10] than their countparts in developed countries. Studies suggest that 50 cases might be necessary for an ophthalmologist to reach proficiency in strabismus surgery [11]. Given these concerns, it is imperative to explore ways to enhance the effectiveness of ophthalmology training programs, ultimately better preparing trainees for performing strabismus surgery.

To drive ophthalmic surgical trainees along the surgical learning curve in a competency-based setup, surgical educators must develop curricula with systematic skill and competency assessments to delegate appropriate responsibilities while ensuring patient safety. However, there is no universally accepted standard for strabismus surgery competency assessment worldwide. Typically, surgical skill is evaluated through the procedure’s constituent steps or phases (e.g., conjunctival incision, exposure of muscle) using videography [12]. Residency training curricula assess surgical steps of intraoperative technical skill based on structured or unstructured rating scales [13–15], which are time-consuming, subjective, and highly variable. Therefore, an automated classification strategy for surgical steps is crucial to enhance trainees’ learning in surgical curricula and serve as an reliable tool for evaluating resident physicians’ performance.

Artificial intelligence (AI), particularly deep learning (DL), has the potential in automated ophthalmic surgical phase recognition. For cataract surgery, Charrière et al. proposed a statistical-based model for real-time analysis cataract surgery videos [16], while Primus et al [17]. adopted DL (recurrent neural network (RNNs)) to automatically assign cataract video frames to operation phases. More recently, our group reported a novel Transformed-based DL algorithm of cataract phase-specific augmented reality (AR) guidance system. Our results demonstrating its superior performance compared to related works [18].

Despite advances in AI-assisted ophthalmic surgical phase recognition, strabismus surgery has received limited attention. This study aimed to develop and validate a novel DL algorithm for automated detecting strabismus surgery steps in the videos without manual intervention. We further compare the performance of the proposed DL algorithm with that of previous RNN-based DL algorithm.

## Methods

### Study design and datasets

In this retrospective cohort study, we collected videos of 496 eyes of strabismus surgery by five pediatric ophthalmologists from the Department of Ophthalmology, Shanghai Children’s Hospital (SCH), between July 2017 and October 2021. The institutional review board (IRB) of SCH approved this study (identifier, 2021R065-F01), and a waiver of informed consent was granted due to the retrospective cohort of deidentified videos captured for training purposes. All methods followed the tenets set forth in the Declaration of Helsinki, and all videos were deidentified according to Health Insurance Portability and Accountability Act (HIPAA) [19].

**Fig. 1:**
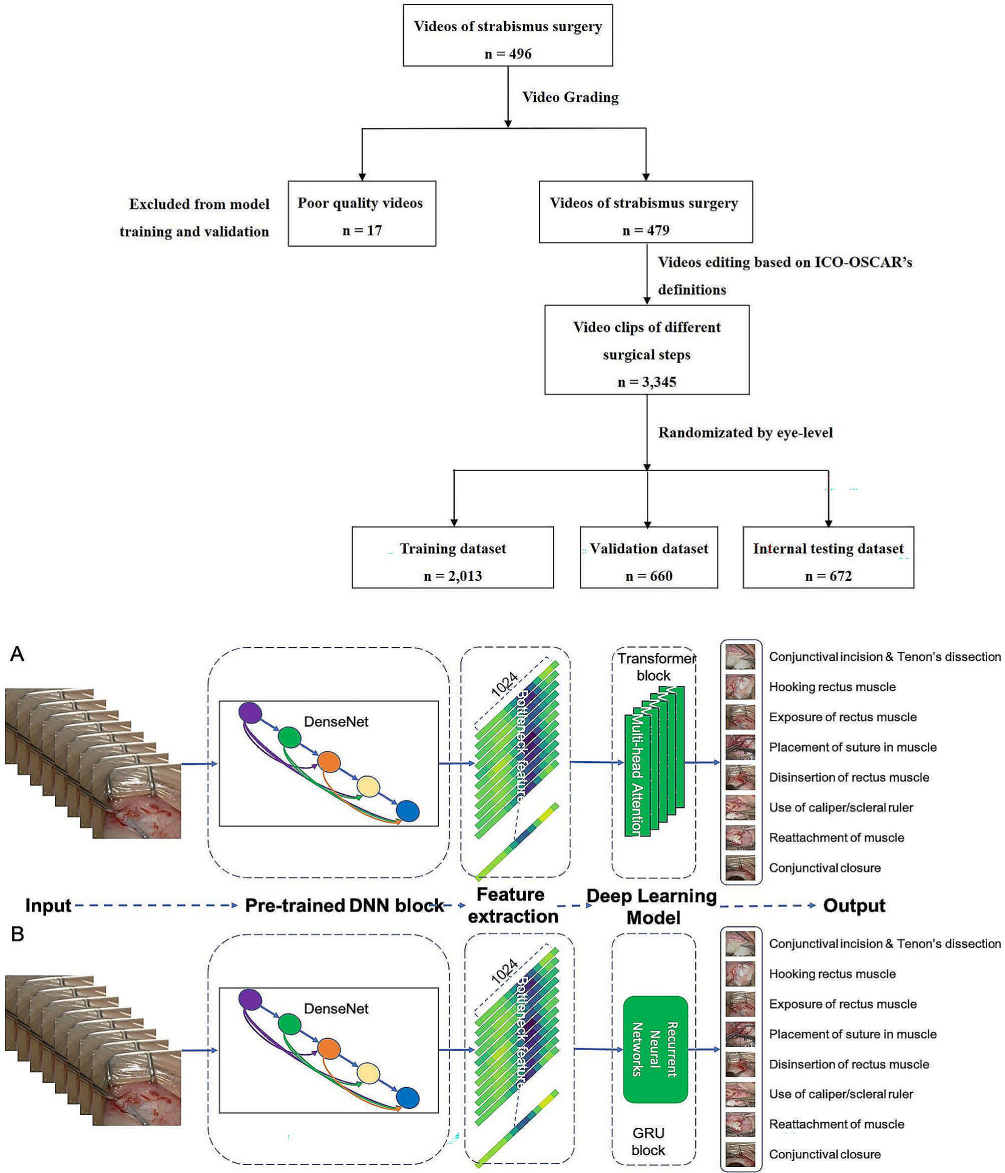
Flowchart of the Current Study and Hybrid DL Algorithm Diagrams. (**A**): a Convolutional Neural Network (CNN) and a Transformer module (RNN-based model) (**B**): a Convolutional Neural Network (CNN) and a Recurrent Neural Network (RNN) consisting of Gated Recurrent Unit (GRU) layers (RNN-based model)

All videos were captured by an integrated digital video-capture system with Zeiss surgical microscopy. Based on the International Council of Ophthalmology’s Ophthalmology Surgical Competency Assessment Rubrics (ICO-OSCAR: strabismus) [20], we analyze eight steps in strabismus surgery: (1) conjunctival incision & Tenon’s dissection, (2) hooking rectus muscle, (3) exposure of rectus muscle, (4) placement of suture in muscle, (5) disinsertion of the rectus muscle, (6) use of caliper/scleral ruler, (7) reattachment of muscle (intrascleral needle pass), and (8) conjunctival closure (when appropriate). Using ICO-OSCAR’s definitions for the eight steps, three pediatric ophthalmologists (XXZ, WYF, XYL and YH) manually identified the start and end of the different surgical steps, and cut the videos into clips. The dataset included 3345 videos of strabismus surgery procedures performed from SCH. We randomly divided the raw video dataset by eye-level into training (for updating model parameters), validation (for hyperparameter tuning and model selection), and testing dataset (for assessing model performance) at a 6:2:2 ratio. The flowchart of the current study was showed in Fig. 1.

### Video processing and development of the DL algorithms

To analyze surgical video, we initially captured frames using OpenCV (Open Source Computer Vision Library. 2015). As videos consist of numerous sequential frames, a single surgical clip may contain thousands of frames that demand expensive hardware. To address this issue, we downsampled the surgical clips to 100 frames using an average method. To enhance our dataset, we performed data augmentation techniques including random cropping and adjustments to saturation, brightness, and contrast.

We evaluated two hybrid DL algorithms (Fig. 1). The first DL algorithm has a CNN and a RNN architecture. To learn spatiotemporal surgical features for the entire step, we use a pre-trained network (DenseNet model) [21] to extract features from the video frames. The DenseNet model is a CNN model, which is a type of DL algorithm that processes images, and is frequently used for diseases classification tasks. We then concatenated these features to train multilayer RNN networks, learning spatiotemporal patterns that discriminate across steps (Fig. 1B). RNN is another type of DL algorithm that process data that comes in a sequence, such as words, sentences, audio, or video [22]. The RNN can learn from the whole sequence of data, not just from individual pieces. For compiling the RNN-based model, we use an Adam optimizer and a minibatch gradient descent of size 32.

The second algorithm we evaluated was a Transformer-based model [23]. Transformer have recently emerged as state-of-the-art DL architectures as described previously by various research groups [24]. Briefly, Transformer architectures are based on a self-attention mechanism that learns the relationships between elements of a sequence. As visual data follows a typical structure (e.g., spatial and temporal coherence), Transformer models and their variants have been successfully used for image recognition [25], object detection [26], and video understanding [27, 28]. Similar to the above RNN-based model, we also adopted a pre-trained DenseNet network for feature extraction. Since videos are ordered sequences of frames, we embed the positions of the frames present inside videos with an Embedding layer (positional encoding) and added these positional embeddings to the precomputed DenseNet feature maps (Fig. 1A). We then applied Transformer with multi-heads networks (number of heads = 6) for video classification. For compiling the Transformer-based model, we use an Adam optimizer (an initial learning rate of 0.001, beta 1 of 0.9, beta 2 of 0.999) and a minibatch gradient descent of size 32. Early stopping was applied when the validation loss did not decrease for ten epochs.

### Experimental setup

We implement the two DL algorithms with the Tensorflow framework (Google, TensorFlow-metal PluggableDevice, version 2.6.0, MacOSX-arm64) and Keras API (version 2.6.0). Our hardware included a MacBook Air (macOS Monterey 12.0.1 operation system) with an Apple M1 chip (7-core GPU and 16-core Neural Engine) and a 16GB RAM system.

### Statistical analysis

According to the Standards for Reporting of Diagnostic Accuracy Studies (STARD) [29], we evaluated the performance of DL algorithms to classify strabismus surgery steps using the following metrics: accuracy, precision, recall and F1-score with a 2-sided 95% confidence interval (CI). Their formulas for calculation are as follows:

1 $$\text{A}\text{c}\text{c}\text{u}\text{r}\text{a}\text{c}\text{y}=\frac{True\, Positive+True\, Negative}{All}$$

2 $$\text{P}\text{r}\text{e}\text{c}\text{i}\text{s}\text{i}\text{o}\text{n}=\frac{True\, Positive}{True Positive+False\, Positive}$$

3 $$\text{R}\text{e}\text{c}\text{a}\text{l}\text{l}=\frac{True\, Positive}{True\, Positive+False\, Negative}$$

4 $$\text{F}1-\text{s}\text{c}\text{o}\text{r}\text{e}=\frac{2*Precision*Recall}{Precision+Recall}$$

We used the area under the receiver operating characteristic (ROC) curve (AUC) to describe the ability of each DL algorithm to discriminate steps of strabismus surgery. For multiclass classification of surgical steps, we used one vs. all technique [30] to estimate steps-specific metrics and their 95% CIs.

We calculate the 95% confidence intervals (CIs) for our model’s performance metrics using the Wilson score interval method. These confidence intervals furnish a statistical measure of certainty regarding the estimates of model performance. Specifically, a narrow confidence interval denotes high confidence in the performance estimate, while a wider interval indicates greater uncertainty. The calculation of the Wilson score interval is as follows:

$$\begin{array}{l}{\rm{CI}}\,{\rm{ = }}\,{\rm{\hat p}}\,{\rm{ \pm }}\,{\rm{z}}\,{\rm{*}}\,{\rm{sqrt((\hat p(1}}\,{\rm{ - }}\,{\rm{\hat p)/n) + (}}{{\rm{z}}^{\rm{2}}}{\rm{/(4}}{{\rm{n}}^{\rm{2}}}{\rm{)))}}\\{\rm{\hat p}}\,{\rm{ = }}\,{\rm{(1/(1}}\,{\rm{ + }}\,{{\rm{z}}^{\rm{2}}}{\rm{/n))}}\,{\rm{*}}\,{\rm{(P}}\,{\rm{ + }}\,{{\rm{z}}^{\rm{2}}}{\rm{/(2n))}}\end{array}$$

Where p̂ represents the model’s performance metrics, z is the z-score corresponding to the standard normal distribution (for a 95% confidence interval, z = 1.96)), and *n* denotes the sample size [31].

All statistical analyses were carried out using Python’s statistical programming language (ver. 3.8.1, Python Software Foundation, Beaverton, US) and sklearn library (ver.1.0.2) [32].

## Results

Of the total 496 strabismus surgical videos, 19 (3.8%) were excluded due to poor video quality or off-center of the surgical area, leaving the total dataset with 479 videos from 249 patients. Among the participants who underwent strabismus surgery, the average age was 6.23 ± 2.91 years, and 121 of them (48.59%) were female.

**Fig. 2:**
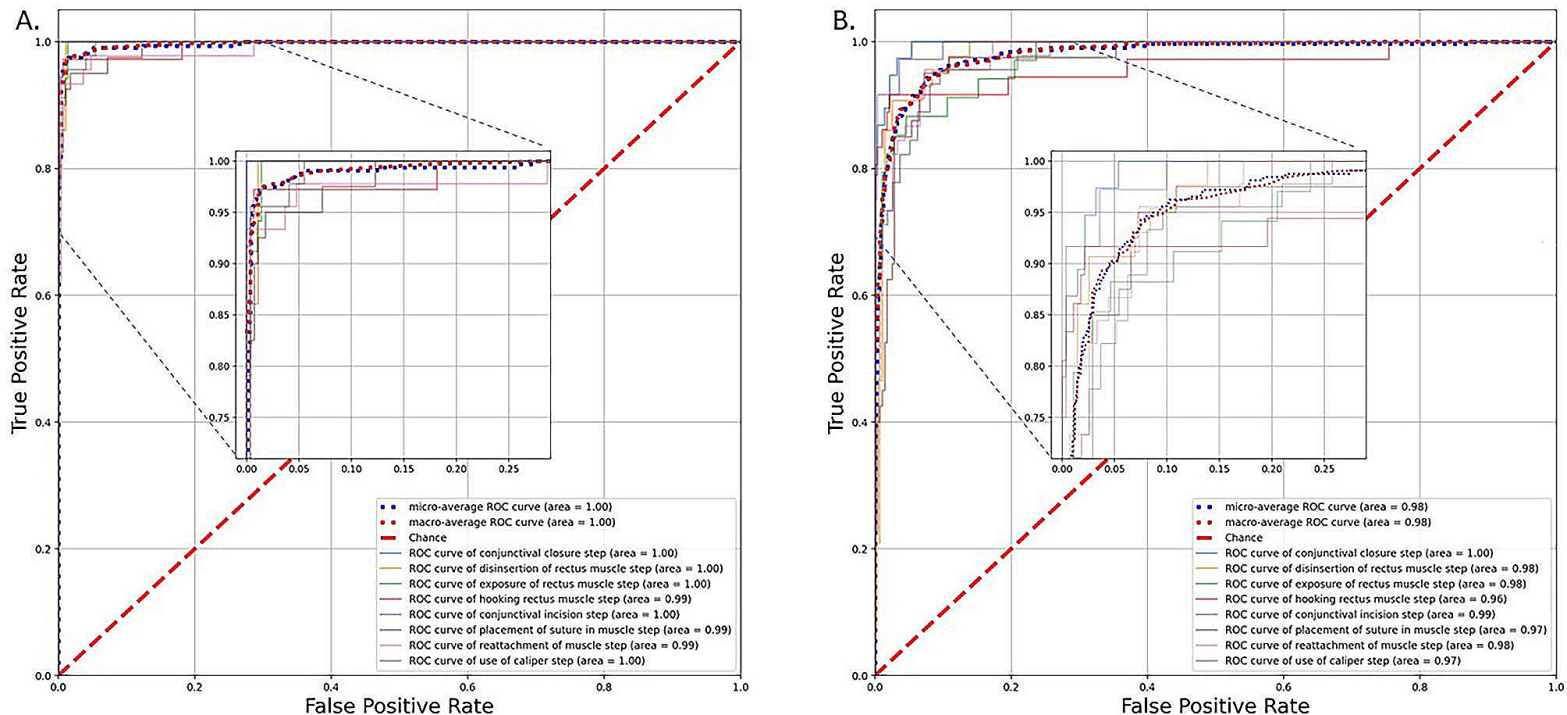
Performance of two DL model in detection of surgical steps in the testing set. (A) ROC curve for detecting different steps of strabismus surgery with the Transformer-based model. (B) ROC curve for detecting different steps of strabismus surgery with the RNN-based model

Supplementary Table 1 illustrates the number of video clips of each step in our dataset. There were 2,013, 660 and 672 video clips of surgical steps in the training, validation and testing dataset. Data augmentation further enriched the sample size of the training dataset to 10,065. After training for 100 epochs (the model showed no improvement in both accuracy and cross-entropy loss, Supplementary Fig. 1), DL models for identifying the steps in video clips of strabismus surgery achieved macro-average AUC of 1.00 (95% CI 1.00–1.00) with Transformer-based model and 0.98 (95% CI 0.97-1.00) with RNN-based model, respectively (Table 1; Fig. 2). Transformer-based model yielded a higher accuracy compared with RNN-based models (0.96 vs. 0.83, *p* < 0.001).

**Table 1:** Summary metrics of algorithm performance for surgical steps classification in validation dataset

Metrics	Transformer-based model	CNN-RNN-based model
Accuracy (95% CI)	0.96 (0.94 to 0.98)	0.83 (0.79 to 0.87)
Macro-AUC (95%CI)	1.00 (1.00 to 1.00)	0.98 (0.97 to 1.00)

**Fig. 3:**
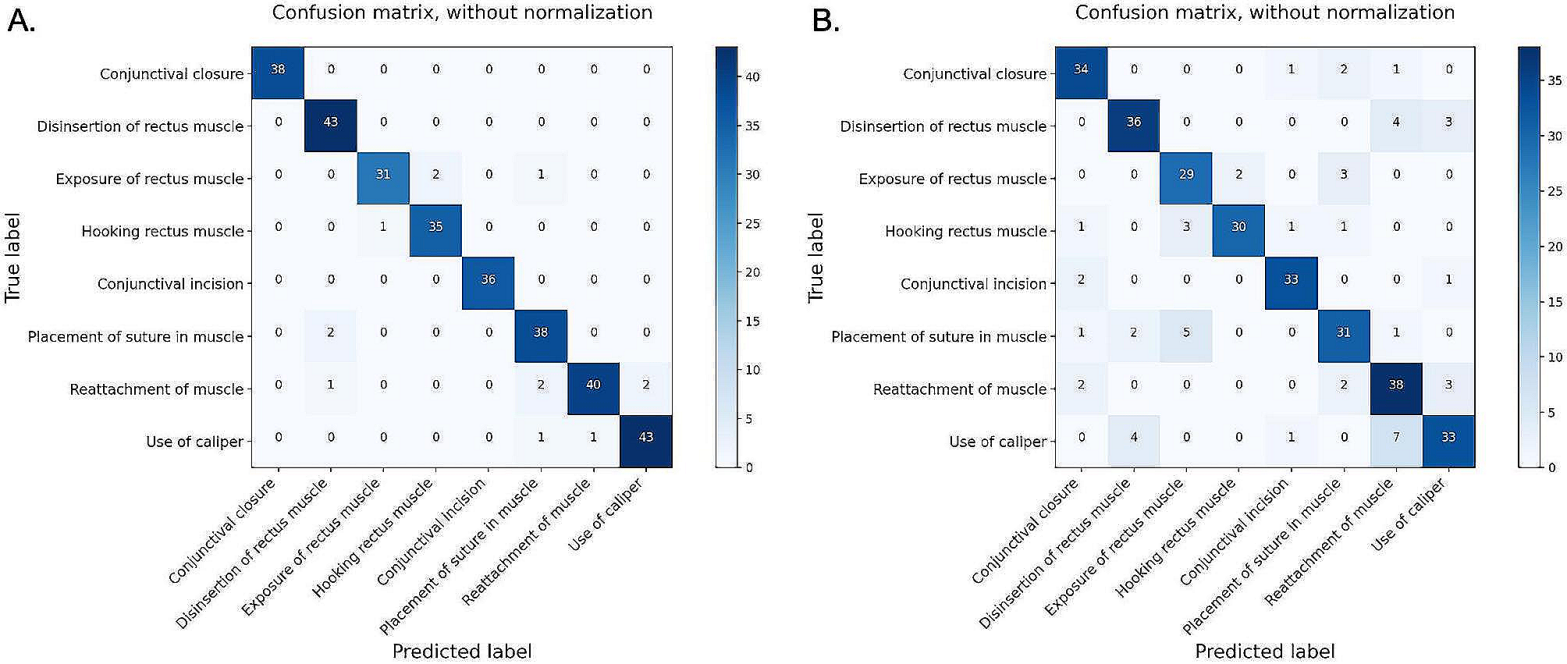
Confusion matrices of two DL model in detection of surgical steps in the testing set. (A)Confusion matrix for detecting different steps of strabismus surgery with the Transformer-based model. (B)Confusion matrix for detecting different steps of strabismus surgery with the RNN-based model

Table 2 demonstrated step-level metrics, including accuracy, sensitivity, specificity, and precision of two DL algorithms across steps. In detecting different steps of strabismus surgery, the predictive ability of the Transformer-based model was better than that of the RNN-based model (Fig. 3). Precision ranged between 0.90 and 1 for the Transformer-based model and 0.75 to 0.94 for the RNN-based model. The f1-score ranged between 0.93 and 1 for the Transformer-based model and 0.78 to 0.92 for the RNN-based model.

**Table 2:** Accuracy, sensitivity, specificity, and precision for algorithms across different steps of strabismus surgery

Algorithm and Metrics	Conjunctival closure	Disinsertion of muscle	Exposure of rectus muscle	Hooking muscle	Conjunctival incision	Placement of suture	Reattachment of muscle	Use of caliper
Transformer-based model								
Accuracy	1	1	0.91 (0.88 to 0.94)	0.97	1	0.95	0.89	0.96
Precision	1	0.93	0.97	0.95	1	0.9	0.98	0.96
Recall	1	1	0.91	0.97	1	0.95	0.89	0.96
F1-score	1	0.97	0.94	0.96	1	0.93	0.93	0.96
CNN-RNN-based model								
Accuracy	0.89	0.84	0.85	0.83	0.92	0.78	0.84	0.73
Precision	0.85	0.86	0.78	0.94	0.92	0.79	0.75	0.82
Recall	0.89	0.84	0.85	0.83	0.92	0.78	0.84	0.73
F1-score	0.87	0.85	0.82	0.88	0.92	0.78	0.79	0.78

## Discussion

In this study, we investigated the performance of the DL algorithms in identifying steps of strabismus surgery from video clips. Our results revealed that the Transformer-based model achieved robust performance (AUC = 1.00; and accuracy = 96%) in classifying different surgical steps. To the best of our knowledge, no other DL system has been developed for recognizing the steps in ophthalmic operation other than cataract surgery. Therefore, we believe that our method provides a unique tool for objective, step-specific assessments of strabismus surgery.

Our study differs from previous cataract surgery studies in several key aspects. First, previous studies primarily relied on CV or machine learning techniques to model instrument labels [33–35], either alone or in combination with video images. In contrast, we adopted a hybrid DL architecture that leveraged Transformer-based models for video classification, building upon our previous work. This approach eliminates the need for instrument segmentation and reduces misalignment and misclassification errors. As limited studies have focused on strabismus surgery, we cannot directly compare our result with state of the art in this field. However, we recently proposed similarly Transformer-based models for cataract phase recognition, which outperforms several strong baselines in surgical phase recognition [36].

Not surprisingly, the Transformer-based model exhibits superior predictive performance compared to the RNN-based model in the current study. RNNs are algorithms for processing sequential data such as natural languages, sound, and time-series data [37]. However, RNNs suffer from gradient explosion/vanishing [38], which makes it challenging to process over long sequences. On the other hand, Transformers are new neural network architectures unveiled by Google AI in 2017 [23]. Utilizing the self-attention mechanism, Transformer-based models capitalize on parallel processing, making the training faster and building a better model in less time. Transformers have outperformed both CNNs and RNNs across a wide range of research areas [39–41]. Our results also confirm that the Transformer-based model demonstrates excellent performance in handling long sequences data. These findings confirm our hypothesis that Transformers are optimal choice for analyzing ophthalmic surgical sequences.

Our study holds the potential in many clinical settings. Ophthalmic surgery, such as strabismus surgery, learning curves for residents are closely tied to feedback-based teaching guidance. A DL algorithm could potentially mitigate surgical errors and guide surgeons, particularly novices, by providing real-time reminders of the next step and warnings for incorrect actions during surgery. It is also possible to develop a real-time supervion and objective surgical evaluation system to improve strabismus surgical outcomes. Wong, et al., recently reported a CNN-based system named DeepSurgery for the evaluation and supervision of cataract surgical procedures [42]. Furthermore, as AI technology advances, intelligent robots equipped with DL algorithms could become invaluable assistants in improving surgical precision and safety. In our previous work, we developed a novel phase-specific augmented reality (AR) [36] guidance system that provides ophthalmologists with customized visual cues based on the recognized surgical phase. This DL algorithm holds the potential to accelerate the development of such intelligent surgical robots, ultimately paving the way for precision medicine in ophthalmology.

There are several limitations to this study. First, our datasets were collected from a single center. There was no external validation dataset to confirm the performance of DL models. Diverse multicenter surgical videos are needed to validate the generalization of our DL model in future studies. Second, we used the dataset with multiple records per surgeon. Further study involving independent datasets with surgeries by different surgeons and clinical contexts is necessary to verify the classification error. Third, we did not include complicated cases or surgery with complications in this pilot study. Therefore, further study is required to assess the generalizability of DL algorithm in complex strabismus surgery cases. Finally, our DL model is developed based on video clips manually labeled and pre-segmented. The real-life applications will require algorithms to detect segment boundaries and assign steps’ labels.

## Conclusion

We demonstrate that DL models can automatically identify strabismus surgery steps with high accuracy based on surgical videos. Furthermore, Transformer algorithms show excellent performance when modeling spatiotemporal features of video frames. Further studies to determine the generalizability of the DL model in real life and its usefulness and potential application in surgical education seem to be warranted.

### Electronic supplementary material

Below is the link to the electronic supplementary material.

Supplementary Material 1

## Data Availability

No datasets were generated or analysed during the current study.
