# Supplementary material for: Automated detection of steps in videos of strabismus surgery using deep learning
Source: BMC Ophthalmol. 2024 Jun 10;24:242. doi: 10.1186/s12886-024-03504-8 (PMC11163806; doi:10.1186/s12886-024-03504-8)
Supplement: Supplementary file 1 — Supplementary Material 1 [file 12886_2024_3504_MOESM1_ESM.docx]

| Supplementary Table 1. Instances of Each Step Within Strabismus Surgery in Dataset | |
| --- | --- |
| Step | Total, No. |
| Conjunctival incision & Tenon’s dissection | 389 |
| Hooking rectus muscle | 389 |
| Exposure of rectus muscle | 388 |
| Placement of suture in muscle | 426 |
| Disinsertion of rectus muscle | 443 |
| Use of caliper/scleral ruler | 448 |
| Reattachment of muscle (intrascleral needle pass) | 454 |
| Conjunctival closure (when appropriate). | 408 |


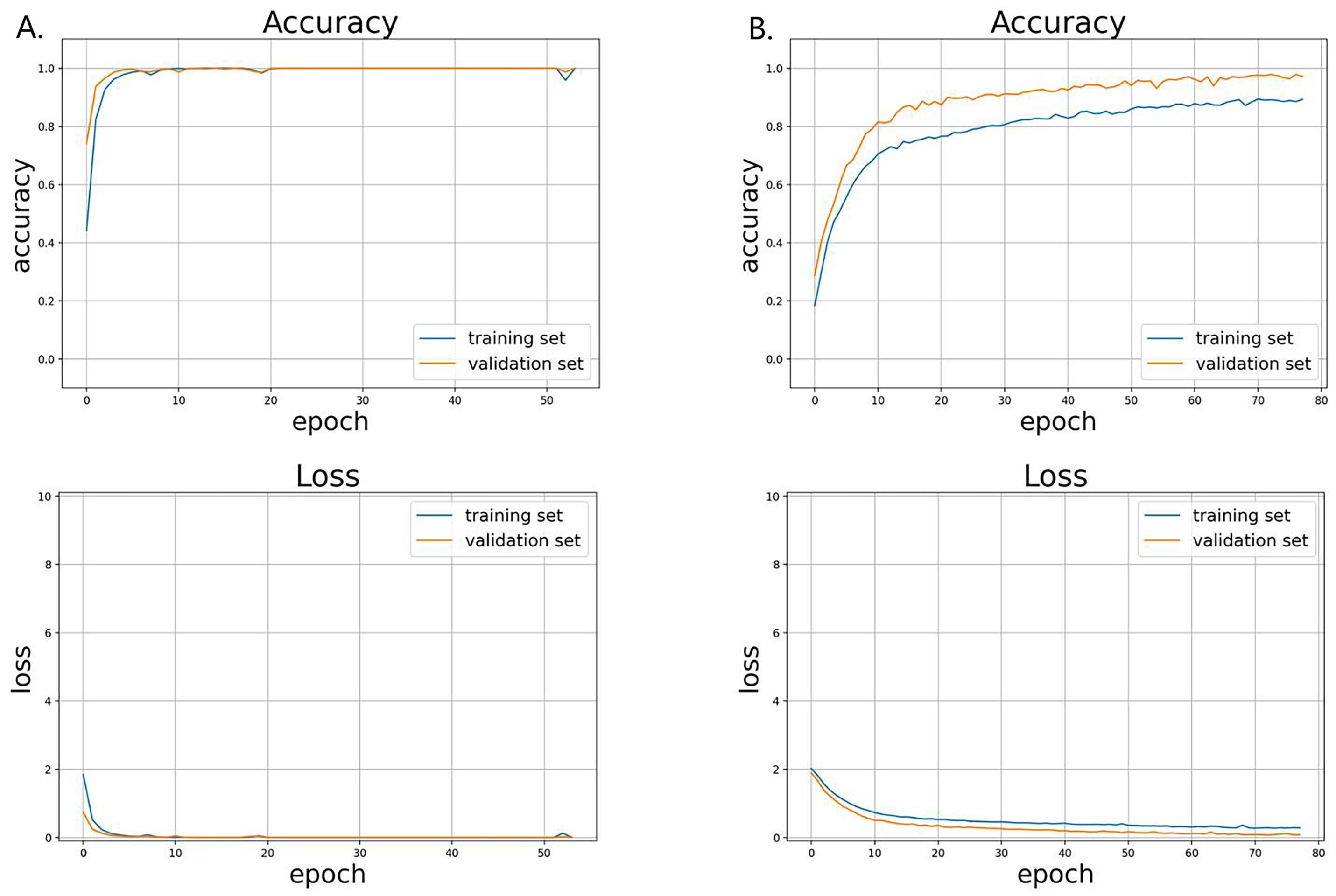


Supplementary Figure 1: Training Dynamics of Two DL Models in Accuracy and Cross-Entropy Loss. (A) Transformer-Based Model. (B) RNN-Based Model
